# Development of Nanosystems Delivering with Eco-Friendly Antibacterial Agents Composed of *Zingiber officinale/Alpinia galanga* and Gold, as Sustainable Antibiotics

**DOI:** 10.3390/pharmaceutics18070894

**Published:** 2026-07-21

**Authors:** Rosa M. Giráldez-Pérez, Elia M. Grueso, Alfonso Carbonero, Antonio Carpintero-López, Rafael Prado-Gotor

**Affiliations:** 1 Department of Cell Biology, Physiology and Immunology, Faculty of Sciences, University of Cordoba, 14014 Cordoba, Spain; b02caloa@uco.es; 2 Department of Physical Chemistry, Faculty of Chemistry, University of Seville, 41012 Seville, Spain; pradogotor@us.es; 3 Department of Animal Health, Veterinary Faculty, University of Cordoba, 14014 Cordoba, Spain; sa1camaa@uco.es

**Keywords:** gold nanosystems, green antibacterial, antibacterial activity, bacteria, *Zingiber officinale*, *Alpinia galanga*

## Abstract

**Background/Objectives:** Green antibacterial synthesis is a natural alternative to chemical methods, aligning with the Sustainable Development Goals by being beneficial to the environment and contributing to new therapies to combat antibiotic resistance. In this research, a simple method was developed for producing nanosystems with natural extracts of Ginger (*Zingiber officinale*) or Galangal (*Alpinia galanga*) with gold. Then, their antibacterial efficacy was evaluated and compared against strains of *Escherichia coli* (*E. coli*) and *Staphylococcus aureus* (*S. aureus*). **Methods:** After optimizing the systems, the core size of the nanosystems was determined using transmission electron microscopy (TEM), and the presence of gold was confirmed with EDS. Stability control was measured using spectrometry, and the systems were characterized by measuring zeta potential and dynamic light scattering (DLS). Finally, to verify the antibacterial effect, minimum inhibitory concentration (MIC) assays were performed. **Results:** The analysis of the MIC experiments confirmed that the Galangal nanosystem (Au@Galangal showed better results against both bacteria. In the case of the Ginger nanosystem (Au@Ginger), the most evident results were against *E. coli*. Internalization studies demonstrated the antibacterial efficacy of all the nanosystems analyzed. **Conclusions:** The results suggest that nanosystems obtained entirely by green methods have great potential as alternative antimicrobial agents, especially against resistant strains, representing a major advance in the development of sustainable bactericidal nanosystems.

## 1. Introduction

Nanotechnology is an emerging and highly relevant field that drives technological innovations with key applications in human biology and medicine, notably advanced treatments and antibiotic delivery [1]. This research area focuses on the analysis and creation of nanomaterials with dimensions ranging from 1 to 100 nanometers [2]. Nanoparticles have generated considerable interest in multiple scientific disciplines, such as medicine [3]. A particularly interesting advantage over other types of conventional materials is that the reduced size of the nanoparticles increases their surface-to-volume ratio. As a result, this specific characteristic allows less material to be used to obtain the same properties as in their macroscopic form, which makes these materials ideal for drug delivery while reducing production costs, in addition to presenting other beneficial properties [4].

Among the various types of nanoparticles, metal nanoparticles stand out as clusters of tiny particles of matter composed of individual atoms, with sizes ranging from 1 to 100 nanometers. Within this type, we find are bare nanoparticles, i.e., those without additional coatings, or nanoparticles with different ligands [5], where the ligand coating the metal core serves to provide protection and stability to the nanoparticles, thus preventing their aggregation [6]. Among the most important metallic nanoparticles are those made of gold, silver, or copper [7]. These have a wide range of applications in the field of medicine due to their physical, chemical, and optical properties [8]. The most notable characteristics of these metallic nanoparticles are their plasmonic, catalytic, and magnetic properties [9]. The plasmonic characteristic is present in metal nanoparticles such as gold, as they have a surface plasmon resonance (SPR) band, which gives them a fundamental optical and photothermal property [10]. On the other hand, their catalytic activity, which is the ability to increase the speed of a chemical reaction [11], stands out as one of the most important chemical applications of this type of nanoparticle. Its efficiency depends on both its morphology and its surface-to-volume ratio, and it can be used both in solution (homogeneous catalysis) and in the solid state (heterogeneous catalysis) [12]. Furthermore, in the specific field of medicine, metal nanoparticles are particularly notable for their remarkable ability to deliver drugs. For instance, magnetic nanoparticles are guided to the target tissue through a magnetic field, thus achieving drug delivery [11]. Therefore, it is not surprising that today, the use of these nanoparticles as antibiotic delivery systems (nanoantibiotics) is gaining great importance. This is because nanoparticles retain the antibiotic much better than if it were applied directly, thus providing stable therapeutic efficacy for longer periods of time [13].

Gold nanoparticles have a wide range of applications in biomedicine and many other fields. Their optical properties, detection capabilities, and ability to transport drugs are particularly noteworthy, and some of these properties can be modified by changing the size, shape, or charge of the nanoparticle [14]. They can be found in the form of nanocubes, nanorods, or nanotriangles, and in different colors depending on their size, thus achieving versatility for a wide range of fields [15]. Surface charge is another very important factor in the uptake and cellular interaction of gold nanoparticles, because modifying the surface charge of the nanoparticles allows them to bind to cell membranes and be internalized within cells more easily, making it an essential process in many biomedical applications [16]. Another key property of gold nanoparticles is their ability to be modified by many agents (which in turn modify their physicochemical properties), thus achieving great results in nanomedicine [17]. Nanoparticles, including gold nanoparticles, can be synthesized using the traditional methods mentioned above. However, the recent synthesis of these nanoparticles from plants is one of the most widely used methods because it avoids the need to handle microorganisms and is much faster and less expensive [18,19]. This synthesis is called green synthesis because it uses plants, bacteria, fungi, or their extracts. It is ecologically friendly, less toxic, and abundant due to the large number of plants that exist, making it a very promising method for synthesis. In fact, in some respects, it can be better than other nanoparticles formed by physical methods [20]. In addition, this type of nanoparticle can be synthesized from plant extracts, thus ensuring that parameters such as pH, temperature, and mixing ratio remain stable, resulting in greater pharmacological activity than those synthesized by other methods [21].

Furthermore, in 2015, all member states of the United Nations adopted the so-called 2030 Agenda for Sustainable Development, thus offering a path to peace and prosperity for people and, therefore, the future. This agenda highlights 17 Sustainable Development Goals (SDGs) as the center of its plan, all of which are important for achieving the intended goal, namely a balance between economic, social, and environmental progress [22]. That is why green synthesis is not only a major advance in nanotechnology but also works in conjunction with the SDGs by connecting technological innovation, sustainability, and public health. Among the SDGs incorporated are the following: SDG 3 (Good Health and Well-Being) by improving the prevention of resistant infections; SDG 9 (Industry, Innovation, and Infrastructure) through the promotion of sustainable technologies in biomedical sectors; SDG 12 (Responsible Consumption and Production) through the optimization of natural resources for nanoparticle synthesis; SDG 13 (Climate Action) by reducing the environmental impact through green synthesis; and SDG 15 (Life on Land) through the integration of toxicology, microbiota, and sustainability studies that strengthen the relationship between human, animal, and environmental health, aligning with the principles of One Health, which responds to global challenges in health and sustainability through advanced scientific approaches (UNDP, n.d.). According to the World Health Organization (WHO), antimicrobial resistance (AMR) [23] represents one of the greatest threats to public health and global progress. It is very important to understand the mechanisms of antimicrobial resistance to develop methods to counteract AMR. The most notable mechanisms are decreased antibiotic absorption (by modifying the external bacterial membrane), modified targets, and antibiotic inactivation by enzymes [24].

Currently, there is great interest in developing different antibacterial treatments from natural products to combat many infections caused by pathogenic microorganisms, which are the main cause of antibiotic resistance [25]. Flavonoids are a broad class of natural products, a series of metabolites that plants produce naturally. They can be found in two forms: aglycone, that is, the active component remaining after the sugar portion has been removed, or in their glycosidic form, which is bound to sugar [26]. In addition, they play an important role in antibacterial activity, as they act through mechanisms different from those of other drugs, enabling them to inhibit nucleic acid synthesis, various functions of the cytoplasmic membrane, and energy metabolism [27]. They also help to reduce adhesion, biofilm formation, and pathogenicity, which promotes bacterial growth [27].

Among distinct flavonoids, this study focuses on the use of Ginger and Galangal as sources of antimicrobial compounds for the preparation of gold nanoparticles. This is because both natural compounds contain high concentrations of bioactive substances capable of attacking bacteria [28].

Ginger (*Zingiber officinale*) is a plant widely used in both gastronomy and medical applications [29]. Ginger contains a phenolic compound called 6-gingerol, which helps combat nausea, pain, and arthritis and has anti-inflammatory and antibacterial properties, among others [30]. 6-gingerol is the most abundant and the one that mainly exhibits the antibacterial property of gingerol; however, there are other gingerols, such as 4-gingerol, 8-gingerol, 10-gingerol, and 12-gingerol [31]. A series of studies has shown that 6-gingerol can inhibit the growth of both Gram-positive and Gram-negative bacteria, among others. Its mechanism of action consists of altering the cell during biofilm formation or interrupting bacterial DNA replication [32].

Galangal (*Alpinia galanga*) is a plant like Ginger, characterized by the presence of compounds such as galangin in its rhizome [33]. Galangin is a flavonol and is characterized by its ability to alter the metabolism of other drugs, being capable of both activating and inhibiting different types of enzymes [34]. In addition, several studies have shown that galangin does not exhibit any cytotoxicity when administered either in vitro or in vivo [35]. Furthermore, galangin has many properties, including anti-inflammatory, antioxidant, antimicrobial, and anticancer properties [36].

In this study, we will focus on two bacteria, *E. coli* (Gram-negative) and *S. aureus* (Gram-positive). *E. coli*, a rod-shaped bacterium belonging to the Enterobacteriaceae family, is found mainly in the gastrointestinal tract in the mucosal layer of the colon [37]. Normally, *E. coli* lives without causing harm; however, it has several pathogenic strains capable of colonizing other places, giving rise to various diseases [38]. These strains can do so thanks to several unique characteristics, such as specific adhesion (enabling them to colonize the small intestine or urethra) [39] and the production of toxic substances [38]. This is why *E. coli* is so pathogenic, as it has managed to create various pathotypes, i.e., it has managed to create groups of strains, causing a disease and a common set of virulent factors for each group [38]. The diseases it can cause can be urinary, gastrointestinal, or even nervous system disorders, the most notable of which are diarrheal disease, urinary tract disease, and sepsis or meningitis [40]. In fact, these strains are not only important because of all the diseases they cause but also because many of them (especially extraintestinal strains) have multiple mechanisms of antibiotic resistance, including beta-lactams and quinolones [41]. *S. aureus* is a type of Gram-positive, spherical, immobile bacterium belonging to the *Micrococaceae* family. Its presence indicates skin contamination [42]. It can invade any organ and cause metastasis through the bloodstream [43]. *S. aureus* is a highly pathogenic bacterium that is widespread and can cause both mild skin infections and much more severe infections [44]. Among the most serious infections it can cause are pneumonia and other respiratory tract infections, among many others. This bacterium is also capable of resisting almost all antibiotics [45] due to its high virulence factors, such as toxin secretion and various immune evasion factors [46].

Although the use of Ginger extract as a reducing agent for gold nanoparticles in eco-friendly synthesis has been reported in previous works [47,48], the use of Galangal is rather scarce. However, to date, research into internalization and colloidal stability over time of green-synthesized nanoparticles continues to address a critical gap in nanotechnology, bridging the safe, eco-friendly synthesis of nanomaterials with their translation into viable, clinical drug-delivery platforms [49]. Thus, while the more recent reports have contrasted green and chemical routes to Ginger-gold nanoparticles [47,48], they were primarily focused on initial characterization and antimicrobial action. Hence, in this context, the main objective of this research is to obtain highly stable gold nanosystems from natural products of Ginger and Galangal extracts using methods compatible with green synthesis, ensuring not only their antibacterial properties against Gram-positive and Gram-negative bacteria but also their stability over time and bacterial internalization. This multifaceted study could help facilitate the implementation and transition of these novel nanosystems from the laboratory to direct clinical application.

## 2. Materials and Methods

### 2.1. Materials

The following is a list of the products used, including the bacteria, along with their product codes and sources: chloroauric acid trihydrate (HAuCl_4_•3H_2_O) from Sigma-Aldrich (St. Louis, MO, USA); organic Ginger (*Zingiber officinale*) from Herbes del Molí, S.L. (Madrid, Spain); cotton muslin bags (reference: ABOHU-50 Organic, Shenzhen, China); Galangal (*Alpinia galanga*) from Naturix24 (Dransfeld, Germany); 96-well U-bottom plates and a Multiskan FC photometer from Thermo Fisher (Waltham, MA, USA); *Escherichia coli* ATCC^TM^ 25922 ^TM^ (reference: R4607050) from Thermo Fisher; *S. aureus* (reference: ATCC^TM^ 29213™) from Thermo Fisher; and resazurin sodium salt (reference: R418900250) from Thermo Fisher. Milli-Q water from Thermo Fisher was used in all the preparations requiring water. OPUS software (version 8.7.10) from Bruker (Billerica, MA, USA).

### 2.2. Methods

#### 2.2.1. Synthesis of Ginger Precursors and Green Nanosystems Formed by Gold Nanoparticles and Ginger (Au@Ginger)

The synthesis of the green Ginger nanosystem (Au@Ginger) was carried out using an “infusion” of powdered Ginger in Milli-Q water, HAuCl_4_, and organic Ginger. First, Milli-Q water (500 mL) was heated in a temperature-controlled stirrer until it reached approximately 80–90 °C. While the water was heating, the Ginger was weighed; in this case, 2.5 g of Ginger was used for every 500 mL of Milli-Q water. Once the Ginger had been weighed, it was placed in a cotton muslin bag measuring approximately 8 × 10 cm for infusion, added to the hot water, and left to infuse for 30 min. After this time, the infused Ginger solution was left to rest. Once cooled, it was filtered a couple of times to remove any possible Ginger residue. After filtering, it was placed in a sonicator for 5 min, after which 50 mL of the solution at pH = 6.39 was extracted and heated to approximately 70 °C. After the precursor was formed, the gold nanosystem was synthesized. First, the solution was heated, and at the same time, a gold solution was prepared, using as a reference that for every 0.02 g of HAuCl_4_, 2 mL of Milli-Q water is dissolved, resulting in a 27.9 mM gold solution. Once the gold solution was made, it was protected with silver foil, and 170 µL was added drop by drop to the 50 mL (0,50% *w*/*v*) of the already heated solution. Thus, the final gold concentration in the mixture was 99.8 nM. Finally, it was left to heat and mix for 10 min and then left to rest, during which time the solution turned pink/reddish in color, indicating that the green gold and Ginger nanosystems (Au@Ginger) had formed. The colloidal mixture was obtained at T = 20.7 °C and pH = 5.59. The observed decrease in the pH of the mixture from the natural gingerol extract to the colloidal solution indicates the successful bio-reduction of gold ions (Au^3+^) to elemental gold (Au^0^) in the formation of Au@Ginger nanoparticles [50].

#### 2.2.2. Synthesis of Galanga Precursors and Green Nanosystems Au@Galangal

After various reaction tests, the optimal amount of Galangal for the formation of the nanosystem was determined. First, 500 mL of Milli-Q water was heated to approximately 80–90 °C. Next, 1.2 g of Galangal, contained in a cotton muslin bag, was added, infused for 30 min, and then left to settle. Once the infusion cooled, it was filtered twice to remove any remaining Galangal. Next, to prepare the nanosystem, the preparation and solution were sonicated for 5 min. Fifty milliliters of the solution at pH = 5.90 were extracted by heating it to 70 °C, and 100 µL of HAuCl4 solution (27.9 mM) was added to 50 mL (0.25% *w*/*v*) of the already heated solution, keeping the reaction in darkness for 10 min. As a result, a stable colloidal mixture was obtained at T = 20.9 °C and pH = 5.01. Again, the decrease in the pH of the mixture from the natural Galangal extract to the colloidal solution indicates the successful bio-reduction of gold ions in the formation of Au@Galangal nanoparticles [50]. The final gold concentration in the mixture was 55.8 nM.

#### 2.2.3. UV/Vis Spectroscopy

Spectroscopy was performed using a Zuzi 4255/50 spectrophotometer (Zuzi, STL DASELAB, S.L., Valencia, Spain). Wavelength measurements were taken in 10 mm quartz cuvettes with continuous recording. The spectral width obtained had an accuracy of ±0.3 nm. To study the stability of the Au@Ginger and Au@Galangal nanosystems, changes in the UV-vis spectra from 400 to 800 nm were followed over time and checked for at least 1 month, following the methodology outlined by Giráldez-Pérez et al., 2020 [51].

#### 2.2.4. Zeta Potential Measurements and Dynamic Light Scattering (DLS)

The functional characterization of the size of the nanosystems was carried out using the dynamic light scattering (DLS) technique. DLS allows particles up to 1 nm in size to be measured and characterized. The sample is irradiated with a laser beam, and, with the help of a photodetector, the fluctuations in the scattered light are recorded at a known scattering angle. DLS can measure the size because the nanoparticles scatter the light, leaving a pattern that can be measured. To complete the characterization, zeta potential was measured using a Zetasizer Nano ZS (Malvern Panalytical, GmbH—Kassel, Germany). This physical property quantifies the magnitude of the surface electric charge of suspended particles and determines the stability of a colloidal system, indicating the repulsive or attractive force between these particles, which depends on the concentration, the ions present in the solution, and the surface charge [52]. In general, based on this technique, a colloid can be considered stable if its zeta potential is greater than about 25 mV [53].

#### 2.2.5. Transmission Electron Microscopy (TEM)

Once the nanosystems had been synthesized, they were observed under a transmission electron microscope to study their shape and size and to perform a microanalysis to demonstrate the presence of gold. For this purpose, a TALOS S200 transmission electron microscope (TEM) (FEI, Seville, Spain) was used. This microscope has a working voltage of 200 kV, can analyze samples at the atomic level, and can obtain images in different modes, such as bright field, annular dark field, and focused dark field. It also allows us to visually observe information about the chemical composition of the sample (HAADF detector) being analyzed, as well as obtain images of transmitted electrons by scanning the beam (STEM). It also has an X-ray detector and single and double rotation sample holders. For observation, a small sample was taken from each nanosystem separately and placed on a copper grid composed of carbon support and left to dry for a few minutes. Once dry, with a magnifying glass and fine tweezers, the copper grid was taken and inserted into the grid holder and then into the TALOS microscope to observe and measure the core of the nanosystems, which is made of gold. From the images obtained in the transmission electron microscope, the size distribution histogram of the Au@Ginger and Au@Galangal nanosystems was constructed using the free software ImageJ 1.54 K program (ImageJ, US National Institutes of Health, Bethesda, MD, USA, https://imagej.net/ij/index.html; accessed on 20 January 2025), thus determining the average size of the nanoparticles. To do this, the photos taken one by one were selected, and the size of each nanosystem was measured using the tools provided by this software based on a known length.

#### 2.2.6. Elemental Microanalysis

EDS, along with FE-SEM, is frequently used to analyze the elemental composition of nanomaterials [54]. The microanalysis was performed using energy-dispersive spectroscopy (EDS) to determine the presence of gold in Au@ginger and Au@Galangal nanosystems, using a high-resolution transmission electron microscope (FEI Company, Hillsboro, OR, USA). To do this, a small sample of each nanosystem was taken separately and placed on a copper grid. To study the elemental components, we prepared bacteria that had been fixed and previously treated with the different treatments.

#### 2.2.7. Fourier Transform Infrared (FT-IR) Analysis

To determine the functional groups responsible for the reduction and stabilization of the nanoparticles, FT-IR (Fourier transform infrared spectroscopy) analysis was performed, an analytical technique that identifies and characterizes chemical compounds and has been applied to the analysis of metal and metal oxide nanoparticles synthesized using environmentally friendly synthesis processes [55]. First, the samples had to undergo freeze-drying to remove H_2_O. To freeze-dry the precursors and nanosystems, they were frozen at a temperature of °C for 2 h. Once external freezing was complete, the freeze-dryer’s condenser cooling was activated. The instrument used was a Telstar freeze-dryer, model Lyoquest-55 freeze-dryer (Albil Testar Technologies S.L.U., Terrassa, Spain), connected to an Ulvac Torricelli-type vacuum pump, model GLD-136C (Ulvac GmbH, Garching bei München, Germany). Next, the samples were placed in the collection ports, and specific vacuum parameters were set. The process began at an initial ambient temperature and atmospheric pressure; the operating temperature was −50 °C, and the pressure was approximately 0.15 mBar. The samples were then freeze-dried by sublimation, a process that took 48 h [56]. FT-IR analysis was performed using an FT-IR spectrometer (Invenio X model, Bruker, Billerica, MA, USA), equipped with a diamond ATR, with a resolution of 4 cm^−1^, 64 sweeps, a sweep range of 4000 to 400 cm^−1^, a MIR source, a TGS RT-DLa detector, a KBr beam splitter, and a sweep rate of 20 kHz. OPUS software (version 8.7.10) Bruker (Billerica, MA, USA) was employed to generate the transmittance curves [55].

#### 2.2.8. Antibacterial Activity of Gold Nanosystems Against Reference Strains

To evaluate the efficacy of our previously studied precursors and nanosystems, 96-well U-bottom plates were used, and tests were performed on *E. coli* and *S. aureus* by means of two-fold microdilution according to the European Committee on Antimicrobial Susceptibility Testing. First, 100 μL of each previously prepared nanosystem and precursor solution was taken into the first well of each row of the plate. Then, 50 μL of Mueller–Hinton medium was added to the second well of each row. Next, 50 μL was taken from the first well of each row and transferred to the second well of each row, thus leaving the product diluted by half. From the second well, it was transferred to the third well of each row and so on until reaching well 6 of each row. The last 50 μL was discarded so that all wells were filled. Finally, 50 μL of bacterial suspension with an optical density of 0.08–0.1 (about 105 CFU) was added to each well. In addition, three corresponding control plates were made on the other half of the plate. The plates were incubated for 24 h in an oven at 37 °C. Next, 30 µL of 0.01% resazurin solution was added to each well, and the plates were incubated again at 37 °C for 4 h to allow the color change to develop; blue wells indicate cell death, while pink wells indicate the presence of live bacteria. After this time, they were transferred to a plate shaker, and the results were read. Each well was read spectrophotometrically in an ELISA reader at a wavelength of 570 nm (Thermo Scientific Multiskan FC Photometer; Waltham, MA, USA). This protocol was repeated three times for each bacterium, such that the results for each well are the average of readings for the three plates. In addition, three “blank” plates were prepared using the same protocol but without the addition of the bacteria in the last step [57].

#### 2.2.9. Obtaining Bacterial Sediment

For the internalization study, bacterial sediment with the tested bacteria was obtained. For this purpose, 1.5 mL Eppendorf tubes, Mueller–Hinton medium, a centrifuge (Eppendorf centrifuge 5804R), 2.5% glutaraldehyde (fixative solution), and 0.1 M cacodylate (washing solution) were used.

First, the different bacterial solutions were prepared for each bacterium from fresh colonies after 24 h of growth. Four 2 mL microcentrifuge tubes were prepared, to which 750 µL of each bacterial solution and another 750 µL of each treatment solution were added. For the negative controls, two additional microcentrifuge tubes were prepared with *E. coli* and *S. aureus* bacterial solutions without any treatment. All samples were gently mixed and incubated for 24 h in an incubator at 37 °C. After this, Eppendorf tubes were gently shaken again and centrifuged for two minutes at 3500 rpm. The supernatant was removed, and 200 µL of glutaraldehyde was added to each tube to fix the sample. Later, they were gently shaken once again and left undisturbed for one hour. Then, they were centrifuged in the same way as before, and the supernatant was removed again. Next, 500 µL of cacodylate (a washing solution) was added to each tube. The tubes were shaken for 10 min to resuspend the pellet and centrifuged again at 3500 rpm for 2 min. This washing step was repeated three times, leaving the last supernatant for further processing. After this, a small pellet was formed at the bottom of the tubes. Finally, these pellets were processed, cut with an ultramicrotome, and observed using a transmission electron microscope, thus revealing the effect that nanosystems have on *E. coli* and *S. aureus* bacteria [57,58].

#### 2.2.10. Internalization of Nanosystems in *E. coli* and *S. aureus*

To observe the internalization of nanosystems in bacteria (from the pellets made in the previous section), a Zeiss Libra 120 electron microscope (Zeiss, Jena, Germany) was used, following protocols outlined by Giráldez-Pérez et al., 2022 [57]. An automatic sample processor was used to process the samples, with a protocol that lasted approximately 34 h. The samples were treated with a 1% osmium tetroxide solution. A 2% uranyl acetate solution was used for contrast and staining the samples, after which the samples were gradually dehydrated, and epoxy resin was added. The samples were then kept at 70 °C for 7 h to polymerize. Once this process was complete, the samples were ready to be cut. After processing the samples, it was necessary to identify the appropriate regions for preparing ultrathin sections. To do this, a preliminary 300 nm cut was made, resulting in semithin sections that allowed us to determine the best regions for study. These samples were examined under a conventional optical microscope after being stained with toluidine blue. Next, using a diamond-tipped knife, ultrathin sections of approximately 70 nm were prepared and mounted on copper grids suitable for analysis on a Zeiss Libra^®^ 120 PLUS transmission electron microscope (TEM) (Zeiss, Oberkochen, Germany).

## 3. Results and Discussion

### 3.1. Stability and Functional Characterization of Au@Ginger and Au@Galangal Nanosystems

**Figure 1:**
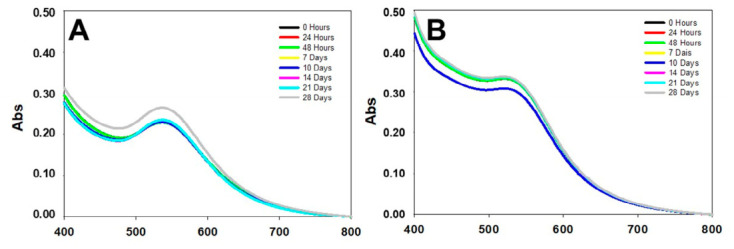
Plot of the stability of Au@Ginger (**A**) and Au@Galangal (**B**).

Stability analysis was performed using UV-vis spectrophotometry over a period of one month and in a wavelength range of 400 to 800 nm. The absorbance curves for Au@Ginger showed an overlap of curves starting at seven days and continuing until the fourth week, with a peak at 536 nm (Figure 1A). In the case of Au@Galangal, the curves were completely superimposed from their synthesis, with a peak at 520 nm (Figure 1B), indicating stability over a month in both cases.

**Figure 2:**
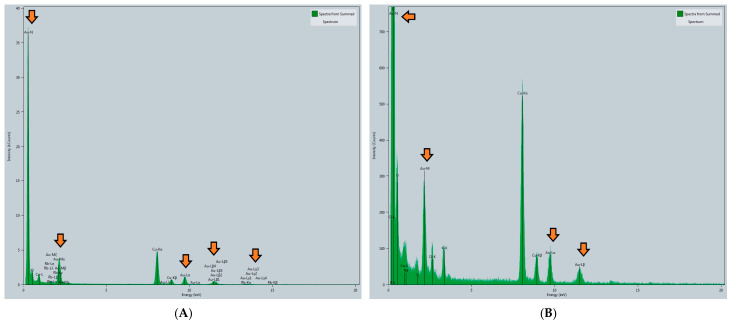
EDS spectrum of the nanoparticles. Elemental mapping results indicate the distribution of gold on the particles. Bear in mind that the presence of copper ions is due to the use of a copper grid coated with a carbon film needed for TEM measurement. Chemical composition of the nanosystems: Au@Ginger (**A**); Au@Galangal (**B**). The arrows indicate the presence of gold.

To confirm the presence of gold in the nanosystems, a microanalysis was performed with the TEM TALOS, where the chemical composition of the samples was observed (Figure 2), confirming the presence of gold in both Au@Ginger (Figure 2A) and Au@Galangal (Figure 2B). The results also showed the presence of other elements such as carbon and copper (due to the composition of the grid) and silicon (due to the microscope detector), among others.

**Figure 3:**
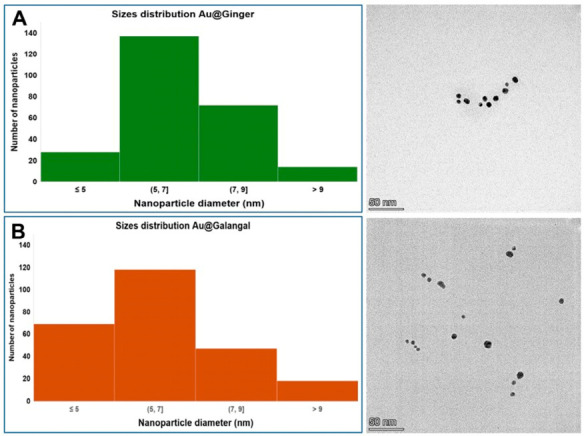
Histogram showing the size distribution of the two nanosystems analyzed. Nanoparticle size distribution using ginger (Au@Ginger) (**A**). Nanoparticle size distribution using Galangal (Au@Galangal) (**B**). On the right are some images obtained using the TALOS TEM.

Size measurement is important when synthesizing nanoparticles because their behavior depends on their size; for example, smaller nanoparticles are able to penetrate bacterial cell membranes much more easily (greater antibacterial activity) than larger nanoparticles [59]. The histograms showing the size measurements of the nanosystems studied are shown below (Figure 3). In this case, the nanosystems were Au@ginger and Au@Galangal.

Analysis of the histograms obtained by TEM showed that the Au@Ginger nanosystems had an average size of (6.6 ± 1.5) nm, while the Au@Galangal nanosystems were slightly smaller, with an average size of (6.1 ± 1.8) nm. However, given the dispersion observed in the size distribution of both nanosystems, it can be concluded that they are very similar in size. Furthermore, their small size, less than 10 nm, combined with the fact that they are clearly monodisperse materials, makes them potentially ideal candidates for antimicrobial treatment.

### 3.2. Functional Characterization

**Figure 4:**
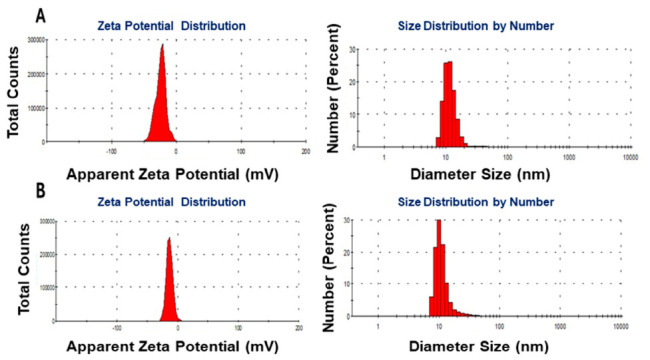
Plots of the zeta potential (**left**) and DLS (**right**) for the Au@Ginger (**A**) and Au@Galangal (**B**) nanosystems.

To complete the functional characterization of the nanosystems, z-potential and DLS measurements were performed (Figure 4).

The following table summarizes the previous figures, providing the exact size and load values for each of the nanosystems studied:

As can be seen from the zeta potential peaks of the two samples (Figure 4) and the summary table (Table 1), the Au@Ginger nanosystem has a value of −27.7 ± 0.7 mV, while the Au@Galangal nanosystem has a value of −13.4 ± 0.8 mV, indicating it tends to form aggregates more easily; however, subsequent stability studies show that they are very stable over the recorded time period.

The size results appear larger than those obtained via the TEM measurements shown in the histograms of Figure 3. The slight differences between the size distribution values obtained by TEM and DLS are primarily due to the fact that TEM measures only the size of the nanoparticle core, whereas DLS estimates the total size of the nanosystem, including the coating agent as well as the contribution of any solvent molecules adhering to its surface [60]. In DLS studies using a Zetasizer to measure particles smaller than 10 nm, difficulties arise due to low scattering intensity, among other factors. The small size of both nanosystems could be due to the fact that both ginger and Galangal contain a large quantity and variety of molecules with reducing capacity, which produce greater nucleation and thus result in a greater number of small nanoparticles.

### 3.3. Fourier Transform Infrared (FT-IR) Analysis of Ginger and Au@Ginger Nanosystem

**Figure 5:**
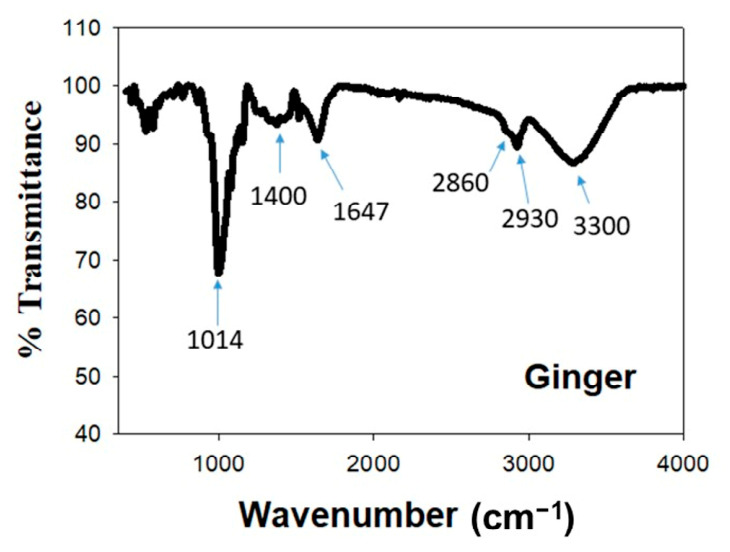
FT-IR spectrum of the Ginger extract.

**Figure 6:**
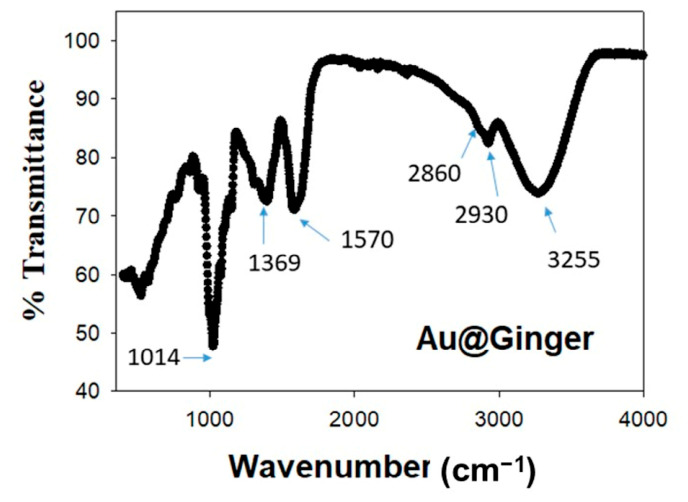
FT-IR spectrum of the ginger-capped gold nanosystem Au@Ginger.

One of the most suitable techniques for identifying the functional groups that likely contribute to the reduction and stabilization of Au@Ginger nanoparticles is FT-IR spectroscopy [61]. Therefore, this technique was used in this study to identify the possible biomolecules present in the nanoparticles that acted as stabilizing and reducing agents during their formation. The Ginger extract’s infrared spectrum, depicted in Figure 5, showed several peaks that correspond to the different functional groups. The peak observed at 3300 cm^−1^ is due to the stretching of the N-H group of the amide and the OH group of the alcohol/phenol, which are present in the aqueous ginger extract (Figure 5). As shown in Figure 6, this peak shifted in the spectrum of the Au@Ginger nanosystem to 3255 cm^−1^, showing a significantly lower transmittance intensity (73.7%) compared to the natural extract (86.6%). A double peak in the C–H stretching spectrum appears at 2930 and 2860 cm^−1^, with intensities of 82% and 84%, respectively, which are almost imperceptible in the Ginger extract. According to the literature, the peaks observed at 1647 and 1400 cm^−1^ in the ginger extract correspond to the C=C band and the C–H bending band, respectively. Both peaks shift to 1570 and 1369 cm^−1^ in the nanoparticle, with a clear decrease in their intensity [61,62,63]. Finally, it is worth noting that the peak observed at 1014 cm^−1^ becomes a prominent band in the nanoparticle. This band can be attributed to C=O stretching vibrations, which are characteristic of polyphenols, alcohols, and ethers. Accordingly, proteins and polyphenolic compounds played an essential role in the formation of the nanoparticle by both stabilizing it and facilitating the reduction of Au^3+^ ions to metallic Au^0^.

### 3.4. Fourier Transform Infrared (FT-IR) Analysis of Galangal Natural Extract and Au@Galanga Nanosystem

**Figure 7:**
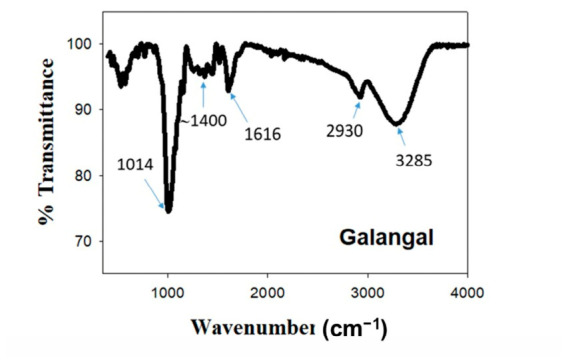
FT-IR spectrum of the Galangal extract.

**Figure 8:**
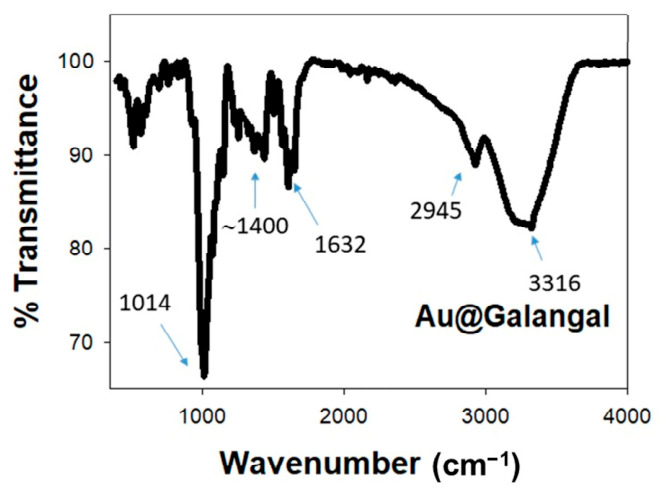
FT-IR spectrum of the Galangal-capped gold nanosystem Au@Galangal.

FT-IR spectroscopy is frequently used to identify the functional groups involved in the reduction and stabilization of nanomaterials derived from plant extracts [64]. The FT-IR spectrum of the pure Galangal extract shows the characteristic bands of its secondary metabolites, where several characteristic bands appeared (Figure 7). By comparing it directly with the FT-IR spectrum corresponding to the Au@Galangal system (Figure 8), it is possible to identify the chemical changes that demonstrate that the synthesis of the nanoparticles has occurred successfully. Taking into account the position of the characteristic IR absorption bands of common organic functional groups [62,64], it can be observed that the H-bonded O–H band of alcohols and phenols in the galangal extract sample at 3285 cm^−1^ shifts to 3316 cm^−1^ in Au@Galangal. This shift is also accompanied by a dramatically lower intensity from 88% to 82%, as shown in Figure 7 and Figure 8. This change indicates a higher concentration at the nanoparticle surface due to confinement or changes in polarity due to bond coordination. A secondary peak is also observed around 2930 cm^−1^, corresponding to the aliphatic C–H stretch, which originates from the carbon chains of the plant’s phytochemicals and shifts slightly to 2945 cm^−1^ as its relative intensity decreases upon nanoparticle formation. In the spectral region ranging from 1400 to 1600 cm^−1^, it can be observed that the small peaks in the extract become more defined and sharper in the sample containing gold. This indicates the oxidation of Galangal upon reduction of the metal. Specifically, the band of medium intensity at 1616 cm^−1^ could be attributed to carbonyl stretching vibrations in the amide bonds of proteins [65,66]; it shifts to 1632 cm^−1^, with a relative decrease in intensity from 93% to 87% transmittance. However, the most dramatic change observed when moving from the extract to the nanoparticle occurs in the band at approximately 1014 cm^−1^, which corresponds to the C–O stretching of the alcohols in the extract. In this case, although the position of the band remains virtually unchanged, its intensity in the pure extract reaches 74%, but in the nanoparticles, it increases significantly to 66%. This confirms that the carbohydrates and alcohols in the extract adhere extensively to the gold surface as stabilizing agents [62,64].

### 3.5. Results of Antibacterial Activity of Gold Nanosystems Against Reference Strains

Once the nanosystems had been characterized, their bactericidal activity was evaluated, beginning with the MIC assay using resazurin staining, in order to determine the minimum concentration required for nanosystems to inhibit the bacteria, in this case, *E. coli* and *S. aureus*. The resazurin microplate double dilution assay was selected, taking into account the existence of some experimental limitations in the conventional colony-forming unit (CFU) agar plating method in the context of nanotechnology-based antimicrobial nanoparticles and natural extracts. That is, nanoparticles often possess distinct optical profiles, induce cell aggregation, or cause local sedimentation, which can compromise the accuracy of standard turbidity measurements in CFU counting and physical colony spacing [67]. For instance, Bélteky and coworkers have reported how silver nanoparticle aggregation caused severe issues in CFU counting by prematurely reducing antimicrobial activity (giving false-positive survival) or by creating false colonies/precipitates on agar plates [68].

**Figure 9:**
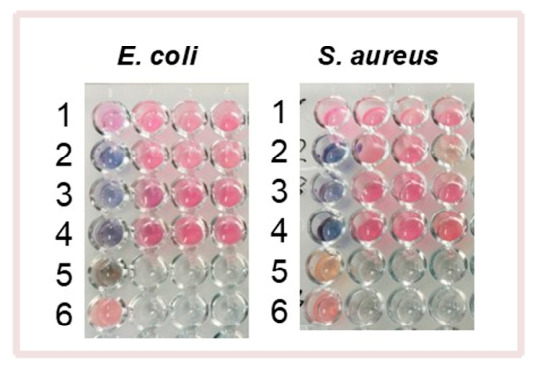
Determination of the minimum inhibitory concentration of the nanosystems and their precursors using the colorimetric microdilution method against *E. coli* and *S. aureus*; 1. Ginger, 2. Au@Ginger, 3. galangal, 4. Au@Galangal, 5. medium without bacteria, and 6. medium with bacteria.

Moreover, the scientific validity and robustness of replacing the CFU method with the resazurin assay are well-established [69]. The results for both bacteria (Figure 9) show complete inhibition at maximum concentration for Au@Ginger and a strong inhibitory effect for Au@Galangal. Noteworthy is the fact that the inhibitory effect of pure Galangal on both *E. coli* and *S. aureus* is almost complete. This evidence can be related to the mechanism of action of pure Galangal, which directly damages both the outer and inner bacterial membranes, leading to cytoplasm coagulation [70]. Note that the blue color is noticeably darker in the case of the functionalized Galangal within the nanoparticle, indicating more significant cell death. However, this effect is not observed with Ginger as the precursor of the Au@Ginger nanosystem (Figure 9).

**Figure 10:**
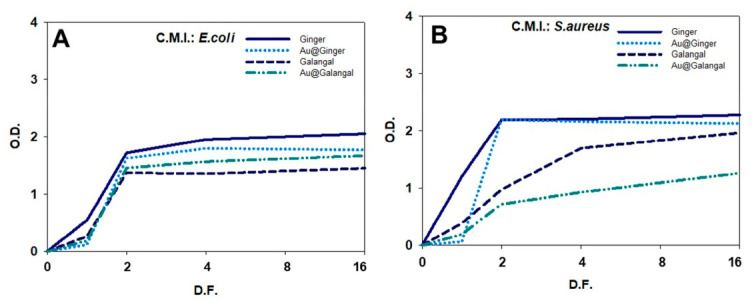
Antimicrobial effect of Ginger and Galangal precursors and their nanosystems (Au@Ginger and Au@Galangal). The optical density measurements are plotted against the dilution factor. (**A**). *E. coli* and (**B**). *S. aureus*. The dilution factor corresponds to 2 to 50%, 4 to 25%, 8 to 12.5%, and 16 to 6.25%.

The graphs showing the antibacterial activity of the different precursors (Ginger and Galangal) and the nanosystems (Au@Ginger and Au@Galangal) confirm that, at 100% concentration, the effects are complete for Au@Ginger, Au@Galangal, and their precursors (Figure 10). The greatest effect on both bacteria is that of Au@Galangal and its precursor, maintaining activity down to a dilution of 6.25%. In summary, we can see that the efficacy of inhibition depends on both the nanosystem and the bacterial strain used. Although all the nanosystems tested exhibited inhibition at their highest concentration, Au@Galangal yielded better results against *S. aureus*, with the effect of its precursor being noticeable in both bacterial strains used. In the case of the Au@Ginger nanosystems, the results were better for *E. coli* at their maximum concentration, offering a new perspective on the effectiveness of using these natural substance carriers as natural antimicrobial agents.

### 3.6. Internalization

**Figure 11:**
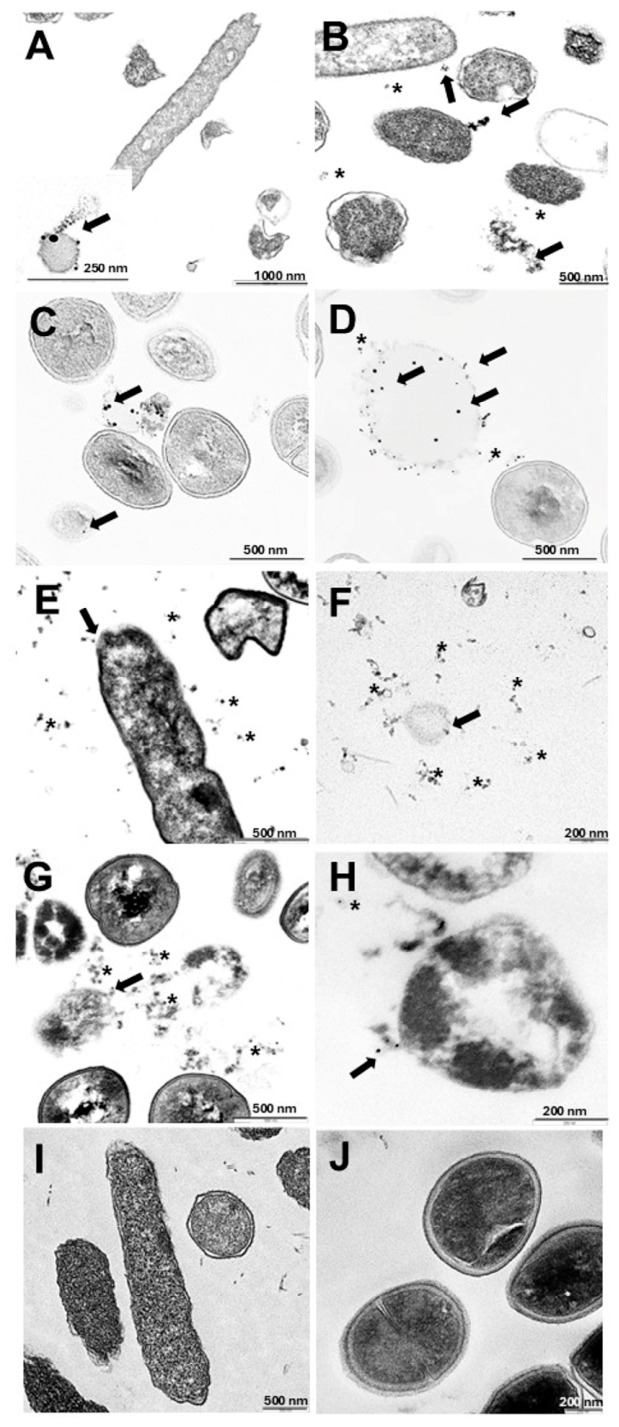
Internalization of nanosystems in *E. coli* (**A**,**B**,**E**,**F**) and in *S. aureus* (**C**,**D**,**G**,**H**), corresponding to Au@Ginger (**A**–**D**) and Au@Galangal (**E**–**H**). The arrows indicate some of the nanosystems interacting with the bacteria, either beginning to enter them or resulting in the destruction of the bacteria. The asterisks indicate the nanosystems present in the vicinity of the bacteria, in some cases along with organic residues. (**I**,**J**) Untreated bacterial populations: (**I**) *E. coli* and (**J**) *S. aureus*.

Studies on the internalization of nanoparticles in bacteria are primarily aimed at developing new antimicrobial treatments, creating nanocarriers for drug delivery, and improving environmental bioremediation processes [71]. In this work, using the pellets that were produced, we conducted a study on the internalization capacity of the nanosystems and their bactericidal properties in both *E. coli* (Gram-negative) and *S. aureus* (Gram-positive) bacteria (Figure 11).

The nanosystems used are negatively charged; therefore, an electrostatic interaction with Gram-positive bacteria may occur. These bacteria lack an outer membrane and have a thick peptidoglycan layer that can reach 100 nm, which contains teichoic acids. Although the lipopolysaccharide in the outer layer of the lipid bilayer has a higher charge per unit area than other phospholipids in Gram-negative bacteria, the interaction of negatively charged nanoparticles with Gram-positive bacteria results in an electrostatic attraction, causing the nanosystems to accumulate on the cell surface and ultimately penetrate the interior of the bacteria. Once inside, the components of the nanosystems trigger a toxicity mechanism by releasing reactive oxygen species and metal ions, in addition to causing protein and enzyme dysfunction, along with the inhibition of signal transduction, including genotoxicity [57,72,73,74,75,76,77,78,79]. Gram-negative bacteria have an additional outer membrane rich in lipopolysaccharides and a thinner layer of peptidoglycan. Therefore, their cell wall has a complex structure, although its thickness is small, ranging from 2 to 10 nm, which makes it easier for nanoparticles to disrupt its integrity by destabilizing the surface and causing the leakage of intracellular substances, leading to bacterial death [57,72,73,74,75,76,77,78,79,80].

Figure 11 shows that some nanosystems reach the vicinity of bacteria (see asterisks). These particles can be internalized through various pathways, such as electrostatic interactions, penetration or membrane damage [57,58,72,73,74,75,76,77,78,79]. As is known in complex biological or environmental media, these nanosystems often exist or interact with surrounding organic residues, which can either facilitate or obstruct their cellular uptake.

In the case of Gram-negative bacteria such as *E. coli*, anionic surface potential nanosystems (Au@Ginger and Au@Galangal) would cause damage to the bacterial surface, allowing them to penetrate the cell and release the gold and the associated compound, causing a disruption in metabolic signal transduction, destroying the microorganism’s biosynthetic machinery, and leading to its death (Figure 11A,B,E,F) [58,73,78]. It appears that this destabilization triggers the production of large amounts of free radicals, which the microorganism is unable to neutralize, contributing to its destruction before it has time to develop resistance [58,73,78]. In the case of Gram-positive bacteria such as *S. aureus*, anionic nanosystems (Au@Ginger and Au@Galangal) accumulate on the cell surface of the microorganism (Figure 11B,C,G,H) due to electrostatic attraction and ultimately penetrate the interior of the bacteria, leading to protein dysfunction through the release of metal ions and enzymes as well as inhibition of signal transduction, including genotoxicity [57,58,72,73,74,75,76,77,78,79]. Therefore, although the internalization mechanism differs between Gram-positive and Gram-negative bacteria, the results obtained show that internalization is effective in both types of nanosystems, confirming that small gold nanoparticles based on both natural Ginger and Galangal products easily penetrate the bacterial cell membrane, leading to its progressive destruction. Finally, Figure 11I–J show untreated populations of *E. coli* and *S. aureus*, revealing the typical morphology of the cells.

## 4. Conclusions

Throughout this study, the results obtained allowed for the successful evaluation of the synthesis and application of green gold nanosystems using simple, precise, and low-cost methods. The Ginger and Galangal nanosystems had small particle sizes, making them suitable for use as antimicrobial agents against bacteria. The Ginger and Galangal nanosystems exhibited anionic surface potential, indicating that their mechanisms of action against Gram-positive and Gram-negative microorganisms are similar.

Stability analyses using UV-vis spectrophotometry showed the high stability of the tested nanosystems, making them products that can be used over time while retaining all their properties.

The efficacy of inhibition depends on both the nanosystem and the bacterial strain used. Although all the nanosystems analyzed showed inhibition at their maximum concentration, Au@Galangal yielded better results against *S. aureus*, with the effect of its precursor being notable in both bacterial strains used. In the case of the Au@Ginger nanosystems, the results were better for *E. coli* at their maximum concentration, offering a new perspective on the efficacy of using these carriers of natural substances as natural antibiotics to combat antibiotic resistance.

Internalization studies demonstrated that the various nanosystems tested are able to penetrate *S. aureus*, a Gram-positive bacterium, due to their electrostatic attraction, whereas in the case of *E. coli*, a Gram-negative bacterium, the negatively anionic nanosystems would cause damage to the bacterial surface. In both cases, this causes disruption of metabolic signal transduction, destroying the microorganism’s biosynthetic machinery and leading to its death. Given the above, while this research into effective and environmentally friendly therapies against antibiotic resistance shows promising results against highly resistant bacteria, it is necessary to evaluate the efficacy of these nanosystems against other resistant bacteria. Furthermore, future studies could assess their effects on complex organisms to verify their effectiveness.

## Figures and Tables

**Table 1:** Particle size analysis using DLS and zeta potential measurement.

Nanosystems	DLS	Zeta Potential
Au@Ginger	10.2 ± 2.8 nm	−27.7 ± 0.7 mV
Au@Galangal	10.1 ± 2.1 nm	−13.4 ± 0.8 mV

## Data Availability

The original contributions presented in this study are included in the article.
